# The APOE^∗^3-Leiden Heterozygous Glucokinase Knockout Mouse as Novel Translational Disease Model for Type 2 Diabetes, Dyslipidemia, and Diabetic Atherosclerosis

**DOI:** 10.1155/2019/9727952

**Published:** 2019-02-21

**Authors:** Marianne G. Pouwer, Suvi E. Heinonen, Margareta Behrendt, Anne-Christine Andréasson, Arianne van Koppen, Aswin L. Menke, Elsbet J. Pieterman, Anita M. van den Hoek, J. Wouter Jukema, Brendan Leighton, Ann-Cathrine Jönsson-Rylander, Hans M. G. Princen

**Affiliations:** ^1^Metabolic Health Research, The Netherlands Organization of Applied Scientific Research (TNO), Gaubius Laboratory, Leiden, Netherlands; ^2^Cardiology, Leiden University Medical Center, Leiden, Netherlands; ^3^Einthoven Laboratory for Experimental Vascular Medicine, Leiden University Medical Center, Leiden, Netherlands; ^4^Cardiovascular, Renal and Metabolism, IMED Biotech Unit, AstraZeneca, Gothenburg, Sweden; ^5^TNO-Triskelion, Zeist, Netherlands; ^6^The Research Network, Sandwich, Kent, UK

## Abstract

**Background:**

There is a lack of predictive preclinical animal models combining atherosclerosis and type 2 diabetes. APOE∗3-Leiden (E3L) mice are a well-established model for diet-induced hyperlipidemia and atherosclerosis, and glucokinase^+/−^ (GK^+/−^) mice are a translatable disease model for glucose control in type 2 diabetes. The respective mice respond similarly to lipid-lowering and antidiabetic drugs as humans. The objective of this study was to evaluate/characterize the APOE^∗^3-Leiden.glucokinase^+/−^ (E3L.GK^+/−^) mouse as a novel disease model to study the metabolic syndrome and diabetic complications.

**Methods:**

Female E3L.GK^+/−^, E3L, and GK^+/−^ mice were fed fat- and cholesterol-containing diets for 37 weeks, and plasma parameters were measured throughout. Development of diabetic macro- and microvascular complications was evaluated.

**Results:**

Cholesterol and triglyceride levels were significantly elevated in E3L and E3L.GK^+/−^ mice compared to GK^+/−^ mice, whereas fasting glucose was significantly increased in E3L.GK^+/−^ and GK^+/−^ mice compared to E3L. Atherosclerotic lesion size was increased 2.2-fold in E3L.GK^+/−^ mice as compared to E3L (*p* = 0.037), which was predicted by glucose exposure (*R* ^2^ = 0.636, *p* = 0.001). E3L and E3L.GK^+/−^ mice developed NASH with severe inflammation and fibrosis which, however, was not altered by introduction of the defective GK phenotype, whereas mild kidney pathology with tubular vacuolization was present in all three phenotypes.

**Conclusions:**

We conclude that the E3L.GK^+/−^ mouse is a promising novel diet-inducible disease model for investigation of the etiology and evaluation of drug treatment on diabetic atherosclerosis.

## 1. Introduction

The metabolic syndrome consists of a cluster of cardiovascular risk factors, including abdominal obesity, elevated blood pressure, elevated fasting plasma glucose, high serum triglycerides, and low high-density lipoprotein (HDL) levels, and drives the global epidemics of type 2 diabetes (T2D) and cardiovascular disease (CVD). Diabetes increases the CVD risk about twofold [1–3], which is the leading cause of death worldwide, and aggravates nonalcoholic steatohepatitis (NASH) [4] and diabetic nephropathy [5]. These comorbidities emphasize the need for antidiabetic treatments that are effective against both T2D and associated cardiovascular complications.

Animal models can be used to learn more about the underlying pathology of diabetic complications and the effect of pharmacological interventions thereon, and a wide range of mouse models combining atherosclerosis and diabetes are described [6]. Most available models are dyslipidemic mice, e.g., ApoE^−/−^ and LDLr^−/−^ mice, with chemically (STZ) or genetically (ob/ob, db/db, and IRS2^−/−^) induced diabetes [6]. Although these models are widely used in biomedical research and drug development, they do not sufficiently reflect human disease. First, deficiency of the *apoe* or *ldlr* gene and STZ treatment result in extreme hyperlipidemia and hyperglycemia, respectively, and may result in overestimation of the contribution of hyperglycemia to diabetic complications. Besides, STZ treatment is difficult to control and creates a type 1 diabetic-like condition. Second, commonly used animal models of T2D (*ob/ob* and *db/db* mice) have a wide but unstable hyperglycemic range [7, 8] and are monogenic models of obesity thereby inducing hyperglycemia, which weakens their translatable value as obesity is seldom caused by a monogenic mutation [7, 9]. Last, ApoE^−/−^ and LDLr^−/−^ mice do not respond well to lipid-lowering drugs used in the clinic [10, 11], making these models unsuitable in the development of novel therapeutic strategies against hyperlipidemia and vascular complications.

The objective of this study was to develop a translational mouse model for the metabolic syndrome and diabetic complications by combining diet-induced dyslipidemia and hyperglycemia, with plasma levels translatable to the human situation: the APOE∗3Leiden.GK^+/−^ mouse (E3L.GK^+/−^). We have generated the E3L.GK^+/−^ mouse by cross-breeding dyslipidemic APOE∗3-Leiden (E3L) mice with hyperglycemic heterozygous glucokinase knockout (GK^+/−^) mice.

The E3L mouse was initially developed as an animal model for mixed dyslipoproteinemia and was generated by the introduction of a DNA construct obtained from a patient with familial dysbetalipoproteinemia (FD) or type III hyperlipoproteinemia containing the human *apoe*∗*3Leiden* and *apoc1* genes [12]. Apoc1 is an inhibitor of lipoprotein lipase (LPL) and inhibits lipolysis of triglyceride-rich lipoproteins. The E∗3-Leiden mutation results in a dysfunctional protein with reduced binding to the low-density lipoprotein receptor (LDLr) which leads to impaired clearance of triglyceride- and cholesterol-rich lipoproteins (chylomicron and VLDL remnants), thereby mimicking the slow clearance observed in humans, particularly in FD patients. E3L mice are prone to develop hyperlipidemia and atherosclerosis upon feeding a Western-type diet containing saturated fat and cholesterol [13], and they respond similarly as humans do to lipid-modulating interventions that are being used in the clinic (e.g., statins, fibrates, niacin, and PCSK9 inhibitors) [11, 14–22].

Glucokinase (GK) catalyzes the first and rate-limiting step in glycolysis, phosphorylation of glucose to glucose-6-phosphate, and acts as a “glucose sensor” in controlling glucose-stimulated insulin secretion [23]. Loss of function mutations in the *Gk* gene in man results in persistent hyperglycemia, referred to as maturity-onset diabetes of the young type 2 (MODY2) [24, 25]. Various transgenic animals with global or tissue-specific GK knockouts have been generated, each with specific characteristics with respect to metabolic control [26]. In this study, we used the global heterozygous GK knockout mouse, which has reduced GK activity in both liver and pancreatic *β*-cells [26]. GK^+/−^ mice are moderately hyperglycemic when on chow, become diabetic on a HFD [26], and respond well to glucose-lowering therapeutic agents (e.g., metformin, sitagliptin, insulin, and exendin-4) [8, 27].

## 2. Materials and Methods

### 2.1. Animals and Breeding

10–23-week-old female E3L, GK^+/−^, and E3L.GK^+/−^ mice (*n* = 6–10/sex/genotype) were used in the study. Both E3L and GK^+/−^ mice are bred on a C57BL/6J background. Since homozygous E3L mice are not viable in utero, these mice are bred heterozygously by breeding E3L X C57BL/6J. GK^+/−^ mice are bred heterozygously (GK^+/−^ X C57bl/6J) as described previously [26], because the homozygous deletion of GK is postnatally lethal. E3L.GK^+/−^ mice were generated by cross-breeding E3L mice with GK^+/−^ mice, thereby generating 27 to 40% offspring of each genotype. Mice were crossed once and were not backcrossed. E3L mice are huApoE3Leiden-huApoC1 double transgenic mice, with both genes located on one genomic DNA construct [12], and therefore, the presence of the E3L phenotype was evaluated by genotyping for ApoC1. The presence of the GK^+/−^ phenotype was evaluated by qPCR as described previously [26]. Females were used because E3L females are more responsive to dietary cholesterol and fat than males. E3L females have a higher VLDL production than males [28] resulting in higher plasma total cholesterol (TC) and triglyceride (TG) levels and development of atherosclerosis [12, 29]. All mice were housed under standard conditions with a 12 h light-dark cycle and had free access to food and water. Body weight was monitored regularly during the study. Animal experiments were approved by the Regional Animal Ethics Committee for Experimental Animals, Göteborg University. All in vivo activities were carried out conforming to the Swedish Animal Welfare Act and regulations SJVFS 2012: 26.

### 2.2. Experimental Design and Analyses

**Figure 1:**
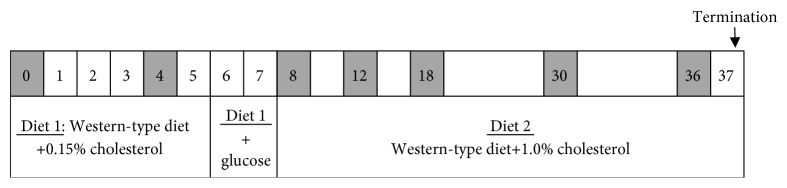
Study design. Female E3L.GK^+/−^, E3L, and GK^+/−^ mice were fed different diets throughout the study. Blood samples were drawn at weeks 0, 4, 8, 12, 18, 30, and 36 as depicted in grey. All mice were sacrificed at week 37. +glucose: 10% glucose drinking water.

First, mice were fed a semisynthetic diet, containing saturated fat with 15% (*w*/*w*) cacao butter (Western-type diet (WTD); Hope Farms, Woerden, the Netherlands) and 0.15% cholesterol for 7 weeks to study the effect of a mildly lipid-elevating diet on plasma lipid and glucose levels. Subsequently, this diet was supplemented with 10% glucose in the drinking water in weeks 6–7 to investigate whether dietary glucose did modulate these plasma levels. During the following 30 weeks, mice were fed WTD+1.0% cholesterol to induce atherosclerosis [20] (Figure 1). EDTA blood samples were drawn after a 4-hour fast, and plasma parameters were evaluated at different time points throughout the study. The last blood sample was drawn at week 36, and all animals were sacrificed by CO_2_ inhalation at week 37. Plasma cholesterol, triglycerides, glucose, and insulin were measured throughout the study, and HbA1c was measured at week 36. Total cholesterol and glucose exposure was calculated by adding up for all intervals the products of the mean cholesterol or glucose level during that interval times the duration of that interval and expressed as mmol/L∗weeks. Lipoprotein profiles, alanine transaminase (ALT), and aspartate transaminase (AST) were measured in groupwise-pooled unfasted sacrifice plasma. Urinary albumin/creatinine levels were measured in spot urine collected in week 36. Hepatic lipid content was analyzed in homogenized, snap-frozen liver samples as described previously [30]. Heart and aorta, liver, and kidneys were collected for histopathological analysis of atherosclerosis, NAFLD/NASH and liver fibrosis, and diabetic nephropathy.

### 2.3. Statistical Analysis

The E3L.GK^+/−^ phenotype was compared to E3L and GK^+/−^, and significance of differences was calculated parametrically using a one-way ANOVA with Dunnett's post hoc test. Differences in plasma parameters between the different time points were calculated for each genotype using a one-way ANOVA with a Bonferroni post hoc test. Significance of differences between the E3L.GK^+/−^ and E3L mice in atherosclerotic lesion number, severity, and composition was calculated using an independent sample *t*-test. A multiple regression analysis was performed to predict the effect of variables on lesion size, and linear regression was used to assess correlations between variables. SPSS 22.0 for Windows was used for statistical analysis. Values are presented as means ± SD. All reported *p* values < 0.05 were considered statistically significant.

For a more detailed description of the applied methods, please see Supplementary Materials online.

## 3. Results

### 3.1. Safety Aspects

No clinical signs of deviant behavior were noted in any of the phenotypes. From weeks 0 to 36, all three phenotypes gained 5 ± 2 gram body weight (Suppl.  Table I). Plasma pooled per group showed lower AST and ALT values as markers of hepatocellular damage in GK^+/−^ mice when compared to E3L.GK^+/−^ and E3L (Suppl.  Table I). One mouse was terminated during the study based on human end point criteria.

#### 3.1.1. Plasma Parameters for Metabolic Disease and Response to Diets

**Figure 2:**
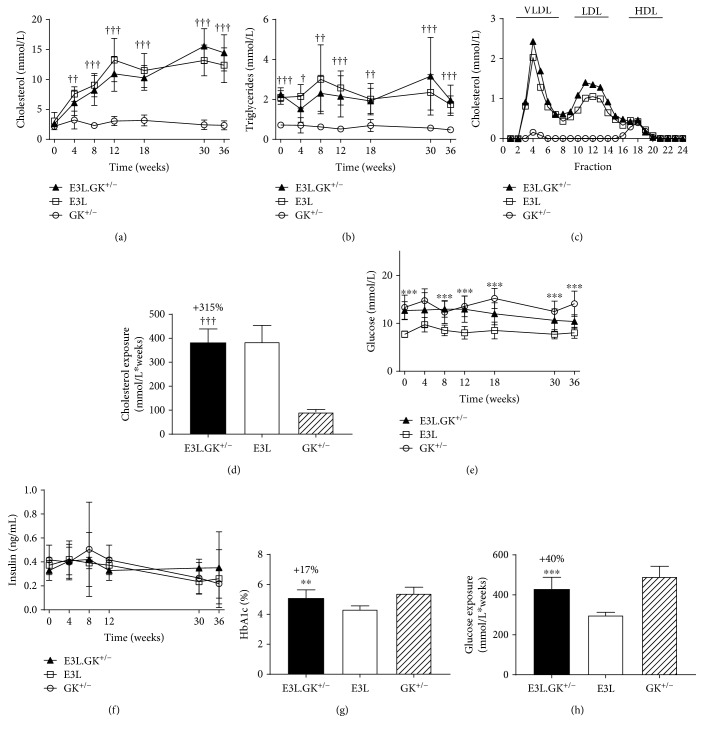
E3L.GK^+/−^ mice have comparable lipid levels and higher glucose levels as E3L mice. Plasma cholesterol (a) and triglycerides (b) were measured throughout the study. Lipoprotein profiles were assessed by FPLC lipoprotein separation in groupwise-pooled unfasted sacrifice plasma (c). Cholesterol exposure over time was calculated as mmol/L∗weeks (d). Plasma glucose (e) and insulin (f) were measured throughout, HbA1c (%) was measured at week 36 (g), and glucose exposure was calculated as mmol/L∗weeks (h). Significance of differences was calculated parametrically using a one-way ANOVA with Dunnett's post hoc test. E3L.GK^+/−^ compared to E3L: ^∗^ *p* < 0.05, ^∗∗^ *p* < 0.01, and ^∗∗∗^ *p* < 0.001; E3L.GK^+/−^ compared to GK^+/−^: ^†^ *p* < 0.05, ^††^ *p* < 0.01, and ^†††^ *p* < 0.001. Data are presented as means ± SD (*n* = 8–10 per group and for insulin *n* = 4–8 per group). FPLC: fast protein liquid chromatography; HbA1c: hemoglobine A1c.

*(1) E3L.GK^+/−^ Mice Are Hyperlipidemic and Hyperglycemic*. Plasma cholesterol and triglyceride levels in the E3L.GK^+/−^ mice were similar to the E3L mice and increased by 540% (cholesterol) and 450% (triglycerides) when compared to the GK^+/−^ mice (Figures 2(a) and 2(b)), resulting in a significantly increased cholesterol exposure (mmol/L∗weeks) (+316%, *p* < 0.001) (Figure 2(d)). Cholesterol in the E3L and E3L.GK^+/−^ mice was mainly contained in VLDL and LDL, and that in GK^+/−^ in HDL (Figure 2(c)). Glucose levels were significantly elevated at all time points except at *T* = 4 weeks when compared to the E3L mice (Figure 2(e)). Total glucose exposure (mmol/L∗weeks) was 429 ± 60, 299 ± 14, and 492 ± 52 mmol/L for E3L.GK^+/−^, E3L, and GK^+/−^, respectively, and significantly increased in E3L.GK^+/−^ when compared to E3L mice (+40%, *p* < 0.001) (Figure 2(h)). Insulin levels did not differ between the strains (Figure 2(f)). HbA1c was increased by 17% when compared to the E3L mice (*p* = 0.005) (Figure 2(g)). In conclusion, these data show that E3L.GK^+/−^ combines both adverse phenotypes with increased lipid levels as in the E3L mice and mildly elevated glucose levels as in the GK^+/−^ mice.

*(2) Plasma Cholesterol Levels Are Modulated by the Diet in E3L.GK^+/−^ and E3L Mice*. Different diets were used in this study to evaluate the response of the mouse model to dietary interventions. Plasma cholesterol, but not triglycerides, increased in both the E3L.GK^+/−^ and E3L mice when switched from a chow diet (*T* = 0 weeks) to a WTD with 0.15% cholesterol added (+143%, *p* = 0.038; +173%, *p* = 0.001), whereas plasma lipid levels were not affected in the GK^+/−^ mice (Suppl.  Table II). Plasma glucose and insulin levels were not affected by the WTD with 0.15% cholesterol added, except for glucose which increased in the E3L mice (+26%, *p* = 0.010). Adding 10% glucose to the drinking water further increased plasma cholesterol levels: when compared to *T* = 0 weeks (chow), cholesterol levels increased by 215% in the E3L.GK^+/−^ mice (*p* = 0.001) and by 224% in the E3L mice (*p* < 0.001). However, this increase was not significant when compared to *T* = 4 (WTD with 0.15% cholesterol) (Suppl.  Table II). Increasing the amount of cholesterol in the diet to 1.0% further increased plasma cholesterol levels in the E3L.GK^+/−^ and E3L mice when compared to *T* = 0 and when compared to *T* = 8 (+89%, *p* < 0.001; +43%, *p* = 0.013). Insulin levels dropped in the E3L mice at *T* = 36 weeks when compared to *T* = 0 weeks (−60%, *p* = 0.020) and *T* = 8 weeks (−66%, *p* = 0.010), whereas this effect was less pronounced in the GK^+/−^ mice (−41%, *p* = 0.081 compared to *T* = 8 weeks) and absent in the E3L.GK^+/−^ mice. Interestingly, plasma glucose levels in the E3L.GK^+/−^ and GK^+/−^ mice were not modulated by glucose in the drinking water, indicating that despite reduced glucokinase activity [26], the mice maintain their glucose homeostasis at an increased glucose supply. Altogether, these data show that plasma lipids can be modulated in the E3L.GK^+/−^ mouse model, as in the E3L mice, whereas the elevated glucose levels on chow are not further increased by these dietary interventions.

#### 3.1.2. Diabetic Macro- and Microvascular Complications in E3L.GK^+/−^ Mice

**Figure 3:**
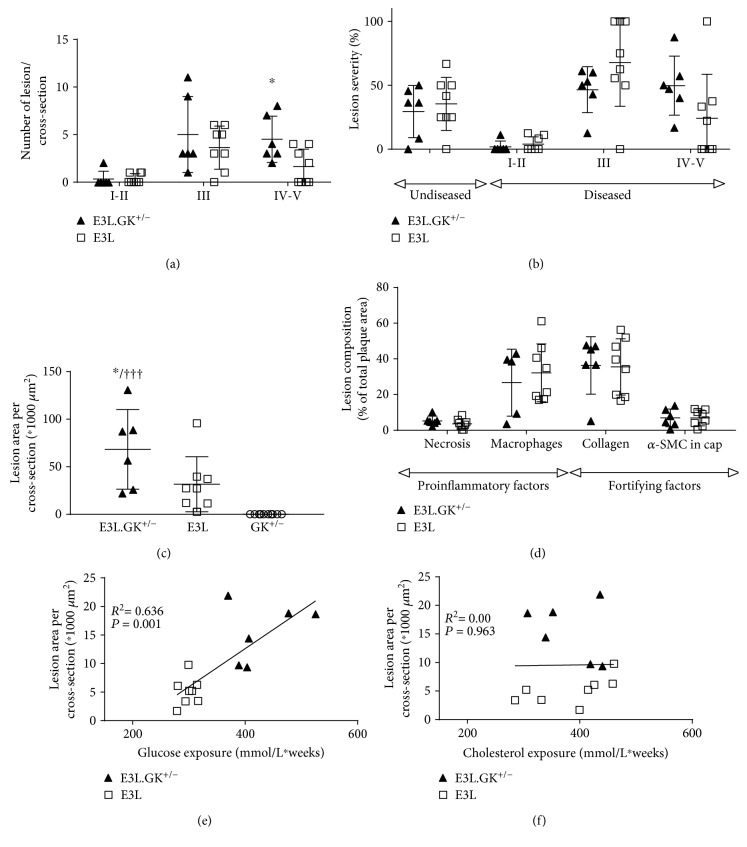
Atherosclerotic lesion size and severity are aggravated in E3L.GK^+/−^ mice which is correlated to glucose exposure. The number of lesions (a); lesion severity classified as mild (type I–II lesions), moderate (type III), and severe (type IV and V) lesions (b); and atherosclerotic lesion size per cross-section (c). Necrotic and macrophage content as proinflammatory factors and *α*SMCs and collagen as fortifying factors were determined in type III–V lesions and expressed as percentage of total plaque area (d). Linear regression analyses were performed on the square root of the lesion area plotted against glucose exposure (e) or cholesterol exposure (f). Significance of differences was calculated two-tailed using an independent sample *t* test (a–d). A multiple regression analysis was performed to predict the effect of variables on lesion size, and linear regression was used to assess correlations between variables (e–f). ^∗^ *p* < 0.05 when compared to E3L; ^†††^ *p* < 0.001 when compared to GK^+/−^. Data are presented as means ± SD (*n* = 6-8 per group).

**Figure 4:**
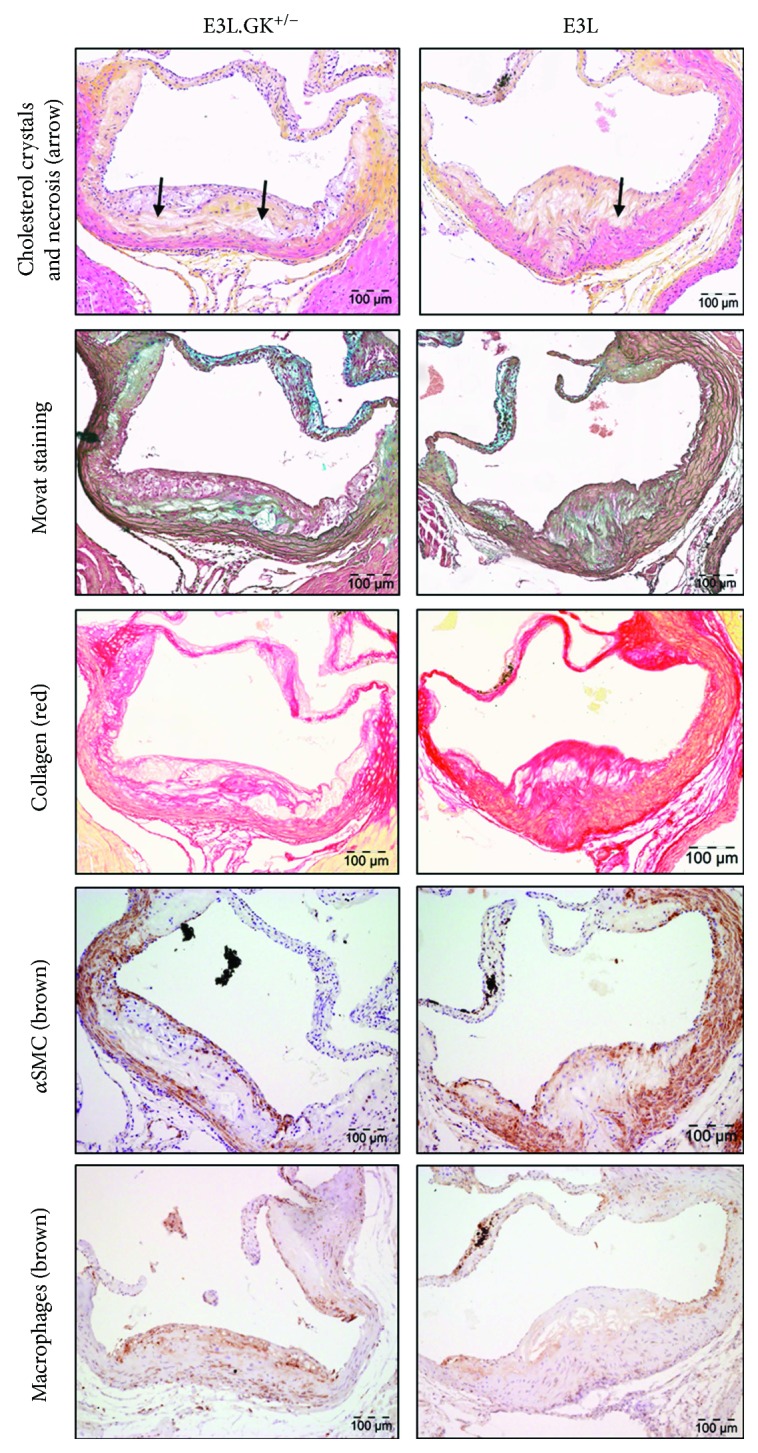
Plaque composition in a severe plaque of a E3L and E3L.GK^+/−^ mouse. Representative images of HPS staining, Movat staining, Sirius Red staining for collagen, immunostaining with *α*-actin for SMCs, and immunostaining with Mac-3 for macrophages. The arrows depict necrotic areas, including cholesterol clefts. HPS: hematoxylin-phloxine-saffron; SMCs: smooth muscle cells.

*(1) Atherosclerotic Lesion Size and Severity Are Aggravated in E3L.GK^+/−^ Mice*. One of the most important diabetic complications is increased risk for CVD [1–3], and therefore, we assessed atherosclerotic lesion size, lesion severity, and plaque phenotype, as markers of vulnerability to rupture, in the aortic root. E3L mice developed 0.4 ± 0.5 mild (I–II), 3.6 ± 2.3 moderate (III), and 1.6 ± 1.8 severe (IV–V) lesions per cross-section. The number of severe lesions was significantly increased in the E3L.GK^+/−^ mice (2.8-fold; *p* = 0.038) (Figure 3(a)). When lesion severity was depicted as the percentage of total plaque area that consisted of mild or severe lesions, there was no difference between the E3L and E3L.GK^+/−^ mice (Figure 3(b)). However, the total atherosclerotic lesion size was significantly increased by 2.2-fold in the E3L.GK^+/−^ mice (68 ± 42∗1000 *μ*m^2^) as compared to E3L (32 ± 29∗1000 *μ*m^2^) (*p* = 0.037) (Figure 3(c)). There were no lesions visible in the GK^+/−^ mice (Figure 3(c)). The plaque composition was analyzed in the type III–V lesions, as illustrated by representative images in Figure 4. There were no significant differences between the E3L.GK^+/−^ and E3L mice in plaque composition (Figure 3(d)), plaque stability index, or monocyte adherence to the endothelium (data not shown). Collectively, these data show that atherosclerotic lesion size is aggravated in E3L.GK^+/−^ as compared to the E3L mice without affecting plaque composition and monocyte adherence.

*(2) Elevated Plasma Glucose Levels Contribute to the Increased Development of Atherosclerosis in E3L.GK^+/−^ Mice*. To explore the contribution of the elevated plasma glucose levels to the increased lesion size, a multiple regression analysis was performed with cholesterol exposure and glucose exposure as covariates after square root transformation of the lesion area. Lesion size was predicted only by glucose exposure (*p* < 0.001). In addition, univariate regression analysis showed a clear association of lesion size with glucose exposure (*R* ^2^ = 0.636, *p* = 0.001) (Figure 3(e)) but not with cholesterol exposure (Figure 3(f)), pointing towards an important role for glucose in the accelerated atherosclerosis development in the E3L.GK^+/−^ mice.

**Figure 5:**
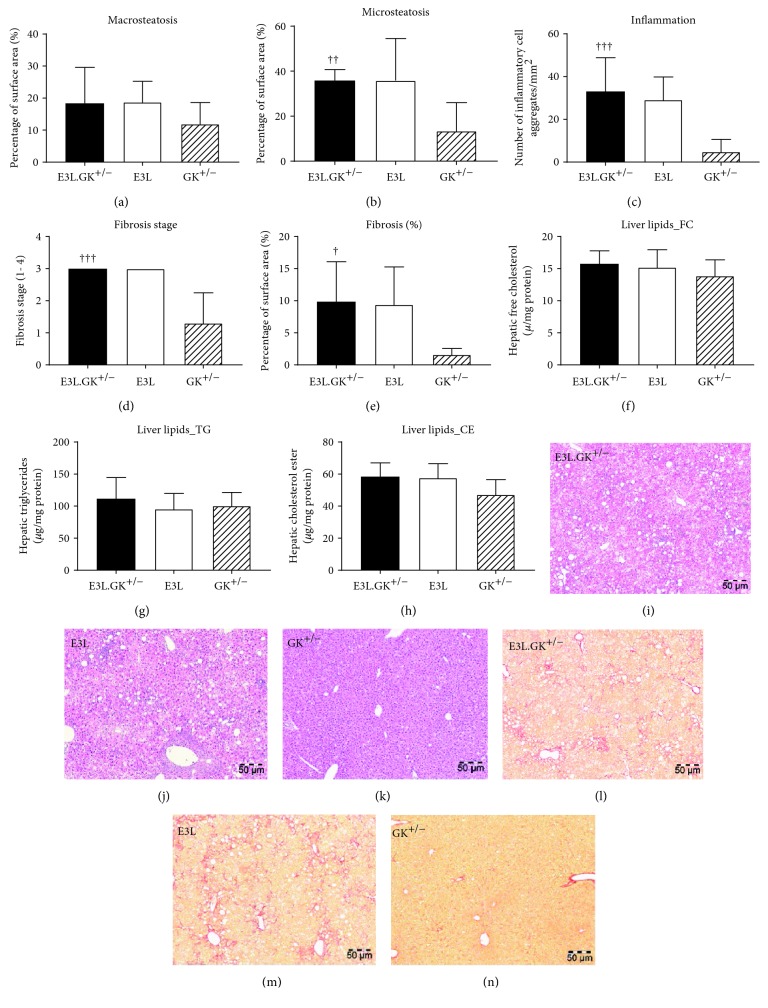
The GK phenotype does not aggravate hepatic steatosis, inflammation, or fibrosis in E3L mice. Macrovesicular steatosis (a) and microvesicular steatosis (b) as percentage of total liver area was determined. The number of inflammatory cell aggregates were counted per mm^2^ (c). Fibrosis grade (1–4) was scored (d) and percentage of area positive for Sirius Red was measured in ImageJ (e). Intrahepatic free cholesterol (f), intrahepatic triglycerides (g), and intrahepatic cholesterol esters (h) were analyzed by HPTLC. Representative images of HE (i–k) and Sirius Red (l–n) staining at a 5x magnification. Significance of differences was calculated parametrically using a one-way ANOVA with Dunnett's post hoc test. ^†^ *p* < 0.05, ^††^ *p* < 0.01, and ^†††^ *p* < 0.001 when compared to GK^+/−^. Data are presented as means ± SD (*n* = 6–10 per group). HPTLC: high-performance thin-layer chromatography; HE: hematoxylin-eosin.

*(3) The GK^+/−^ Phenotype Does Not Aggravate Hepatic Steatosis, Inflammation, or Fibrosis*. NAFLD/NASH is strongly associated with the metabolic syndrome and type 2 diabetes [31, 32]. To assess whether the GK^+/−^ phenotype worsens the development of NASH, liver sections were examined for hepatic steatosis, inflammation, and fibrosis, and liver lipid content was measured. Hepatic macrosteatosis did not differ between the phenotypes (Figure 5(a)), whereas hepatic microsteatosis was significantly elevated by 2.7-fold (*p* = 0.003) in the E3L.GK^+/−^ mice when compared to GK^+/−^ (Figure 5(b)), and both E3L.GK^+/−^ and E3L had severe liver inflammation which was increased by 6.8-fold (*p* < 0.001) in E3L.GK^+/−^ relative to GK^+/−^ (Figure 5(c)). Furthermore, mean fibrosis stage in E3L.GK^+/−^ was significantly elevated when compared to GK^+/−^ (2.3-fold, *p* < 0.001) (Figure 5(d)), as well as the percentage Sirius Red-positive area of total liver area (6.1-fold, *p* = 0.011) (Figure 5(e)). Liver lipids did not differ between the phenotypes (Figures 5(f)–5(h)). Representative images are shown (Figures 5(i)–5(n)). Collectively, these data show that the E3L and E3L.GK^+/−^ mice, but not GK^+/−^, develop NASH with severe inflammation and fibrosis, which is not worsened by increased glucose levels. This indicates a dominant role for the combination of the E3L phenotype and dietary cholesterol in the progression of NASH and liver fibrosis.

**Figure 6:**
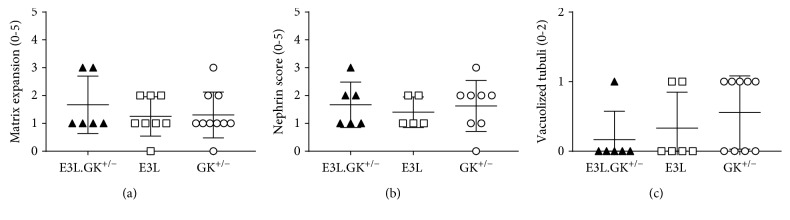
Mild matrix expansion and vacuolized tubuli in all phenotypes. Presence of matrix expansion (a), nephrin score (b), and vacuolized tubuli (c) was scored in a range of 0–5. Significance of differences was calculated parametrically using a one-way ANOVA with Dunnett's post hoc test. Data are presented as means ± SD (*n* = 7–10 per group).

*(4) Mild Kidney Pathology Is Present in All Three Phenotypes*. Diabetic nephropathy is becoming an increasingly important cause of morbidity and mortality worldwide and is related to the increasing prevalence of type 2 diabetes. Therefore, kidneys were analyzed for the presence of renal damage focusing on glomerular damage, including mesangial matrix expansion, and tubulointerstitial damage, including interstitial inflammation, fibrosis, and tubular abnormalities, as central causes for loss of kidney function. Nephrin staining was performed to study renal filtration barrier function. There were no differences in inflammation, fibrosis (data not shown), mesangial matrix expansion (Figure 6(a)), or nephrin score (Figure 6(b)) between the phenotypes. Abnormal tubular structures were observed in all three phenotypes but were most pronounced in the GK^+/−^ mice, wherein the tubuli showed vacuolization (Figure 6(c)). The pathological changes did not affect permeability in the glomerulus, as measured by the urinary albumin : creatinine ratio (Suppl.  Table I). Altogether, we can conclude that mild pathological changes are present, which are not aggravated in the E3L.GK^+/−^ mice.

## 4. Discussion

In the present study, we evaluated the E3L.GK^+/−^ mouse as an animal model for diet-induced hyperlipidemia and hyperglycemia and the pathological consequences thereof. We showed that plasma lipids can be titrated to desired and for human relevant levels by adding cholesterol and fat to the diet and that these levels remain stable for a long period (up to 37 weeks). In addition, the E3L.GK^+/−^ mice were mildly hyperglycemic and developed more atherosclerosis than the E3L mice, which was related to the higher glucose levels in the E3L.GK^+/−^ mice. The E3L and E3L.GK^+/−^ mice both developed hepatic steatosis with severe inflammation and fibrosis, which, however, was not altered by introduction of the defective GK phenotype, whereas only mild kidney pathology with tubular vacuolization was present in all three phenotypes.

Translatability of animal models is essential when investigating the pathogenesis of diabetic complications and evaluating drug treatment thereon. Plasma cholesterol and glucose levels in the diet-induced E3L.GK^+/−^ mouse model were similar to levels in patients with increased cardiovascular risk [2, 33]. Partial deletion of the *Gk* gene in the E3L mice did not affect the response of plasma lipids to dietary modulation, and in both the E3L.GK^+/−^ and E3L mice, plasma cholesterol levels raised similarly upon feeding a WTD with increasing amounts of cholesterol. Interestingly, glucose and insulin levels were not affected by the diet but remained stable representing mild hyperglycemia in the E3L.GK^+/−^ and GK^+/−^ mice (10.4 ± 1.4 mmol/L and 14.1 ± 2.6 mmol/L at the end point, respectively). In contrast, glucose levels in male GK^+/−^ mice increase over time on a high-fat diet with plasma levels reaching 18.9 ± 1.0 mmol/L and impaired glucose tolerance [8, 26]. This gender difference may be explained by the C57BL/6J background of the E3L and GK^+/−^ transgenic mice. Upon a high-fat diet, insulin and glucose levels increase over time in C57BL/6J males, consistent with insulin resistance and glucose intolerance, whereas C57BL/6J females have normal serum insulin concentrations and glucose levels remain constant [34]. Estrogens affect different metabolic pathways in the glucose hemostasis [35], thereby protecting against the risk of developing type 2 diabetes in both premenopausal women [36] and mice [35].

We observed a markedly increased atherosclerotic lesion size in E3L.GK^+/−^ as compared to the E3L mice which was highly significantly correlated with glucose exposure (*R* ^2^ = 0.636, *p* = 0.001), suggesting a proatherogenic role of glucose in the development of atherosclerosis. Indeed, it is known that prolonged exposure to hyperglycemia negatively affects the endothelium, vascular smooth muscle cells, and macrophages, and it increases thrombosis while impairing fibrinolysis, leading to formation of atherosclerotic plaques [37]. This may explain the association of diabetes type 2/hyperglycemia with cardiovascular disease as found in both humans [1, 2, 37–40] and hyperglycemic mice [6], including the E3L.GK^+/−^ mice.

In the present study, both the hyperglycemic GK^+/−^ mice and the hyperlipidemic E3L and E3L.GK^+/−^ mice developed hepatic steatosis, in line with the pathogenesis of NAFLD wherein both metabolic overload and hyperlipidemia contribute to the accumulation of triglycerides and cholesterol in the liver. Interestingly, E3L and E3L.GK^+/−^, but not GK^+/−^ mice, developed extensive inflammation and hepatic fibrosis, pointing towards a role for cholesterol in the transition of NAFLD to NASH. Consistent with this view, when cholesterol is supplied to HFD diet, E3L mice develop NASH and liver fibrosis as well [41], and E3L and E3L.CETP mice have been shown to be established diet-induced NASH and liver fibrosis models [41, 42]. In a previous study with E3L mice, an increased amount of hepatic cholesterol crystals was found and intrahepatic free cholesterol levels were positively correlated with the number of inflammatory aggregates and the expression of hepatic proinflammatory and profibrotic genes [43]. Similarly, it has been shown that accumulation of free cholesterol leading to the formation of cholesterol crystals in hepatocyte lipid droplets may trigger the progression of simple steatosis to NASH both in patients and in mice [44]. Since no additional effects of glucose were observed on hepatic inflammation or fibrosis in the E3L.GK^+/−^ mice, we suggest that hyperlipidemia rather than hyperglycemia is an initiator of hepatic inflammation and fibrosis.

Chronic kidney disease is a largely irreversible disease characterized by tubulointerstitial inflammation, fibrosis, and glomerulosclerosis. The present study describes only mild kidney pathology without microalbuminuria in all three phenotypes. In addition to risk factors investigated in this study (hyperglycemia and dyslipidemia), hypertension plays a central role in renal injury through increasing renal tubular reabsorption and causing a hypertensive shift of renal-pressure natriuresis [5]. Studies on nephropathic patients showed that decreased blood pressure reduced the incidence of renal events and improved kidney function [45, 46]. In the present study, blood pressure was not measured. However, it is known that the E3L mice do not develop hypertension upon a WTD but do respond to antihypertensive treatment [15, 17], and although there are no reports in the GK^+/−^ mice, glucokinase deficiency in humans does not aggravate blood pressure [25].

Previously, the GK^+/−^ApoE^−/−^ mouse model has been developed as a model combining hyperlipidemia and hyperglycemia, which had impaired glucose tolerance and a minimal increase of atherosclerosis relative to ApoE^−/−^ mice [47]. A disadvantage of this model is the ApoE^−/−^ background. ApoE^−/−^ mice are, like LDLr^−/−^ mice, a severe model for hyperlipidemia, and due to the absence of a functional apoE-LDLr-mediated clearance pathway, these mice do not respond well to lipid-lowering drugs (e.g., statins [10], PCSK9 inhibitors [11]) and therefore cannot be used for the evaluation of combination treatment. In contrast, the E3L mice are very suited to study lipoprotein metabolism and lipid modulation [10, 48].

**Figure 7:**
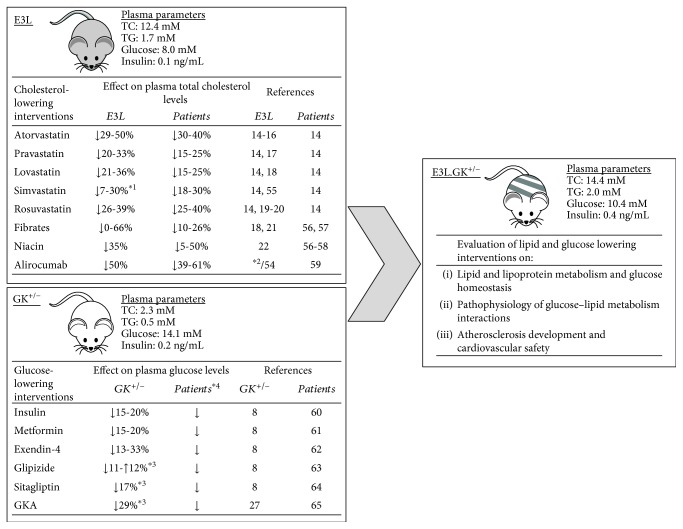
Overview of intervention studies with cholesterol- and glucose-lowering drugs performed in the E3L and GK^+/−^ mouse models. The effects of cholesterol-lowering interventions on plasma total cholesterol levels were evaluated in E3L mice in long-term (5–28 weeks) exposure studies. The effects of glucose-lowering interventions on free-feeding blood glucose profiles were evaluated in GK^+/−^ mice after single or repeated^∗^^3^ dosing. In all studies, mice were fed a high-fat or high-fat/cholesterol-containing diet. Data are extrapolated from published studies (see References). The depicted plasma parameters were measured at the end point in the present study. ^∗1^Data shown for both APOE^∗^3-Leiden and APOE∗3-Leiden.CETP mice. ^∗2^Unpublished. See Reference [54] for data obtained from APOE∗3-Leiden.CETP mice. ^∗3^Repeated dosing. ^∗4^As doses in diabetic patients are generally adapted to reach the desired plasma glucose level of <8 mM, reductions are not depicted as percentages. TC: total cholesterol; TG: triglycerides; GKA: glucokinase activator [54–65].

In Figure 7, we give an overview of all registered cholesterol- and glucose-lowering drugs that have been evaluated in the E3L and GK^+/−^ mice, respectively. The E3L mice respond similarly as humans do to lipid-lowering agents, including statins, fibrates, niacin, and PCSK9 inhibitors [11, 14–22], whereas glucose levels are successfully reduced in the GK^+/−^ mice by standard therapeutic agents as insulin, metformin, exendin-4, and GKAs at doses corresponding to therapeutic drug levels in man [8, 27]. Although these interventions have not been assessed in the E3L.GK^+/−^ mice yet, we carefully speculate about the effects and discuss how the model can be of value for future research. As the E3L.GK^+/−^ mice have similar lipid and glucose levels as their parent models and respond in a similar way to dietary modulations, we propose that both lipid- and glucose-lowering agents will be effective in the combined model. Also, we propose that the E3L.GK^+/−^ mice can be used to examine interactions between glucose and lipid metabolism, e.g., how statin treatment increases the risk of diabetes incidence [49]. Last, atherosclerosis development and cardiovascular safety can be evaluated in the E3L.GK^+/−^ model, which is especially interesting regarding the currently unknown mechanisms by which glucose-lowering agents (e.g., empagliflozin, liraglutide, and semaglutide) improve CV outcome [50–52].

## 5. Conclusion

Altogether, we conclude that the E3L.GK^+/−^ mouse is a promising translatable diet-inducible model, combining dyslipidemia and hyperglycemia with human-like plasma cholesterol and glucose levels and aggravated atherosclerosis, to study the etiology of diabetic atherosclerosis and for the evaluation of lipid-lowering and antidiabetic drugs and their combination thereon.

## Data Availability

The data used to support the findings of this study are available from the corresponding author upon request.

## Supplementary Materials

Supplementary Materials The Supplementary Materials consist of (1) a detailed description of the applied methods and (2) 2 tables: Table I and Table II. Suppl. Table I: biochemical parameters in E3L.GK^+/−^, E3L, and GK^+/−^ mice. All depicted parameters are measured at week 36, except for liver weight (week 37). Suppl. Table II: plasma cholesterol levels are modulated by the diet in E3L.GK^+/−^ and E3L mice. The response of plasma lipids, glucose, and insulin to the different diets was evaluated.

## References

[1] Barengo N. C., Katoh S., Moltchanov V., Tajima N., Tuomilehto J. (2008). The diabetes-cardiovascular risk paradox: results from a Finnish population-based prospective study. *European Heart Journal*.

[2] The Emerging Risk Factors Collaboration (2010). Diabetes mellitus, fasting blood glucose concentration, and risk of vascular disease: a collaborative meta-analysis of 102 prospective studies. *The Lancet*.

[3] Bae J. C., Cho N. H., Suh S. (2015). Cardiovascular disease incidence, mortality and case fatality related to diabetes and metabolic syndrome: a community-based prospective study (Ansung-Ansan cohort 2001-12). *Journal of Diabetes*.

[4] Tarantino G., Saldalamacchia G., Conca P., Arena A. (2007). Non-alcoholic fatty liver disease: further expression of the metabolic syndrome. *Journal of Gastroenterology and Hepatology*.

[5] Maric C., Hall J. E. (2011). Obesity, metabolic syndrome and diabetic nephropathy. *Contributions to Nephrology*.

[6] Heinonen S. E., Genové G., Bengtsson E. (2015). Animal models of diabetic macrovascular complications: key players in the development of new therapeutic approaches. *Journal of Diabetes Research*.

[7] Lindstrom P. (2007). The physiology of obese-hyperglycemic mice [*ob/ob* mice]. *The Scientific World Journal*.

[8] Baker D. J., Atkinson A. M., Wilkinson G. P., Coope G. J., Charles A. D., Leighton B. (2014). Characterization of the heterozygous glucokinase knockout mouse as a translational disease model for glucose control in type 2 diabetes. *British Journal of Pharmacology*.

[9] King A. J. F. (2012). The use of animal models in diabetes research. *British Journal of Pharmacology*.

[10] Zadelaar S., Kleemann R., Verschuren L. (2007). Mouse models for atherosclerosis and pharmaceutical modifiers. *Arteriosclerosis, Thrombosis, and Vascular Biology*.

[11] Ason B., van der Hoorn J. W. A., Chan J. (2014). PCSK9 inhibition fails to alter hepatic LDLR, circulating cholesterol, and atherosclerosis in the absence of ApoE. *Journal of Lipid Research*.

[12] van den Maagdenberg A. M., Hofker M. H., Krimpenfort P. J. (1993). Transgenic mice carrying the apolipoprotein E3-Leiden gene exhibit hyperlipoproteinemia. *The Journal of Biological Chemistry*.

[13] van Vlijmen B. J., van den Maagdenberg A. M., Gijbels M. J. (1994). Diet-induced hyperlipoproteinemia and atherosclerosis in apolipoprotein E3-Leiden transgenic mice. *The Journal of Clinical Investigation*.

[14] van de Steeg E., Kleemann R., Jansen H. T. (2013). Combined analysis of pharmacokinetic and efficacy data of preclinical studies with statins markedly improves translation of drug efficacy to human trials. *The Journal of Pharmacology and Experimental Therapeutics*.

[15] Delsing D. J. M., Jukema W. J., van de Wiel M. A. (2003). Differential effects of amlodipine and atorvastatin treatment and their combination on atherosclerosis in ApoE^∗^3-Leiden transgenic mice. *Journal of Cardiovascular Pharmacology*.

[16] Verschuren L., Kleemann R., Offerman E. H. (2005). Effect of low dose atorvastatin versus diet-induced cholesterol lowering on atherosclerotic lesion progression and inflammation in apolipoprotein E^∗^3–Leiden Transgenic Mice. *Arteriosclerosis, Thrombosis, and Vascular Biology*.

[17] van der Hoorn J. W. A., Kleemann R., Havekes L. M., Kooistra T., Princen H. M. G., Jukema J. W. (2007). Olmesartan and pravastatin additively reduce development of atherosclerosis in APOE^∗^3Leiden transgenic mice. *Journal of Hypertension*.

[18] van Vlijmen B. J., Pearce N. J., Bergö M. (1998). Apolipoprotein E^∗^3-Leiden transgenic mice as a test model for hypolipidaemic drugs. *Arzneimittel-Forschung*.

[19] Delsing D. J. M., Post S. M., Groenendijk M. (2005). Rosuvastatin reduces plasma lipids by inhibiting VLDL production and enhancing hepatobiliary lipid excretion in ApoE^∗^3-Leiden mice. *Journal of Cardiovascular Pharmacology*.

[20] Kleemann R., Princen H. M. G., Emeis J. J. (2003). Rosuvastatin reduces atherosclerosis development beyond and independent of its plasma cholesterol–lowering effect in APOE^∗^3-Leiden transgenic mice: evidence for antiinflammatory effects of rosuvastatin. *Circulation*.

[21] Kooistra T., Verschuren L., de Vries-van der Weij J. (2006). Fenofibrate reduces atherogenesis in ApoE^∗^3Leiden mice: evidence for multiple antiatherogenic effects besides lowering plasma cholesterol. *Arteriosclerosis, Thrombosis, and Vascular Biology*.

[22] van der Hoorn J. W. A., de Haan W., Berbée J. F. P. (2008). Niacin increases HDL by reducing hepatic expression and plasma levels of cholesteryl ester transfer protein in *APOE*
^∗^
*3Leiden.CETP* mice. *Arteriosclerosis, Thrombosis, and Vascular Biology*.

[23] Postic C., Shiota M., Niswender K. D. (1999). Dual roles for glucokinase in glucose homeostasis as determined by liver and pancreatic *β* cell-specific gene knock-outs using Cre recombinase. *Journal of Biological Chemistry*.

[24] Fajans S. S., Bell G. I., Bowden D. W., Halter J. B., Polonsky K. S. (1996). Maturity onset diabetes of the young (MODY). *Diabetic Medicine*.

[25] Velho G., Blanché H., Vaxillaire M. (1997). Identification of 14 new glucokinase mutations and description of the clinical profile of 42 MODY-2 families. *Diabetologia*.

[26] Gorman T., Hope D. C. D., Brownlie R. (2008). Effect of high-fat diet on glucose homeostasis and gene expression in glucokinase knockout mice. *Diabetes, Obesity and Metabolism*.

[27] Baker D. J., Wilkinson G. P., Atkinson A. M. (2014). Chronic glucokinase activator treatment at clinically translatable exposures gives durable glucose lowering in two animal models of type 2 diabetes. *British Journal of Pharmacology*.

[28] van Vlijmen B. J., van 't Hof H. B., Mol M. J. (1996). Modulation of very low density lipoprotein production and clearance contributes to age- and gender- dependent hyperlipoproteinemia in apolipoprotein E3-Leiden transgenic mice. *The Journal of Clinical Investigation*.

[29] Trion A., de Maat M. P. M., Jukema J. W. (2005). No effect of C-reactive protein on early atherosclerosis development in apolipoprotein E^∗^3-Leiden/human C-reactive protein transgenic mice. *Arteriosclerosis, Thrombosis, and Vascular Biology*.

[30] Post S. M., Zoeteweij J. P., Bos M. H. (1999). Acyl-coenzyme A:cholesterol acyltransferase inhibitor, avasimibe, stimulates bile acid synthesis and cholesterol 7*α*-hydroxylase in cultured rat hepatocytes and in vivo in the rat. *Hepatology*.

[31] Kotronen A., Yki-Järvinen H., Männistö S. (2010). Non-alcoholic and alcoholic fatty liver disease - two diseases of affluence associated with the metabolic syndrome and type 2 diabetes: the FIN-D2D survey. *BMC Public Health*.

[32] Lim H. W., Bernstein D. E. (2018). Risk factors for the development of nonalcoholic fatty liver disease/nonalcoholic steatohepatitis, including genetics. *Clinics in Liver Disease*.

[33] The Emerging Risk Factors Collaboration (2009). Major lipids, apolipoproteins, and risk of vascular disease. *JAMA*.

[34] Pettersson U. S., Walden T. B., Carlsson P. O., Jansson L., Phillipson M. (2012). Female mice are protected against high-fat diet induced metabolic syndrome and increase the regulatory T cell population in adipose tissue. *PLoS One*.

[35] Louet J. F., LeMay C., Mauvais-Jarvis F. (2004). Antidiabetic actions of estrogen: insight from human and genetic mouse models. *Current Atherosclerosis Reports*.

[36] Crespo C. J., Smit E., Snelling A., Sempos C. T., Andersen R. E., NHANES III (2002). Hormone replacement therapy and its relationship to lipid and glucose metabolism in diabetic and nondiabetic postmenopausal women: results from the Third National Health and Nutrition Examination Survey (NHANES III). *Diabetes Care*.

[37] Laakso M., Kuusisto J. (2014). Insulin resistance and hyperglycaemia in cardiovascular disease development. *Nature Reviews Endocrinology*.

[38] Ross S., Gerstein H. C., Eikelboom J., Anand S. S., Yusuf S., Pare G. (2015). Mendelian randomization analysis supports the causal role of dysglycaemia and diabetes in the risk of coronary artery disease. *European Heart Journal*.

[39] Ahmad O. S., Morris J. A., Mujammami M. (2015). A Mendelian randomization study of the effect of type-2 diabetes on coronary heart disease. *Nature Communications*.

[40] Roussel R., Steg P. G., Mohammedi K., Marre M., Potier L. (2018). Prevention of cardiovascular disease through reduction of glycaemic exposure in type 2 diabetes: a perspective on glucose-lowering interventions. *Diabetes, Obesity and Metabolism*.

[41] Liang W., Verschuren L., Mulder P. (2015). Salsalate attenuates diet induced non-alcoholic steatohepatitis in mice by decreasing lipogenic and inflammatory processes. *British Journal of Pharmacology*.

[42] Zimmer M., Bista P., Benson E. L. (2017). CAT-2003: a novel sterol regulatory element-binding protein inhibitor that reduces steatohepatitis, plasma lipids, and atherosclerosis in apolipoprotein E^∗^3-Leiden mice. *Hepatology Communications*.

[43] Morrison M. C., Liang W., Mulder P. (2015). Mirtoselect, an anthocyanin-rich bilberry extract, attenuates non-alcoholic steatohepatitis and associated fibrosis in ApoE^∗^3Leiden mice. *Journal of Hepatology*.

[44] Ioannou G. N., Haigh W. G., Thorning D., Savard C. (2013). Hepatic cholesterol crystals and crown-like structures distinguish NASH from simple steatosis. *Journal of Lipid Research*.

[45] Bakris G. L., Williams M., Dworkin L. (2000). Preserving renal function in adults with hypertension and diabetes: a consensus approach. *American Journal of Kidney Diseases*.

[46] de Galan B. E., Perkovic V., Ninomiya T. (2009). Lowering blood pressure reduces renal events in type 2 diabetes. *Journal of the American Society of Nephrology*.

[47] Adingupu D. D., Heinonen S. E., Andréasson A.-C. (2016). Hyperglycemia induced by glucokinase deficiency accelerates atherosclerosis development and impairs lesion regression in combined heterozygous glucokinase and the apolipoprotein E-knockout mice. *Journal of Diabetes Research*.

[48] Princen H. M. G., Pouwer M. G., Pieterman E. J. (2016). Comment on “Hypercholesterolemia with consumption of PFOA-laced Western diets is dependent on strain and sex of mice” by Rebholz S.L. et al. Toxicol. Rep. 2016 (3) 46–54. *Toxicology reports*.

[49] Sattar N., Preiss D., Murray H. M. (2010). Statins and risk of incident diabetes: a collaborative meta-analysis of randomised statin trials. *The Lancet*.

[50] Zinman B., Wanner C., Lachin J. M. (2015). Empagliflozin, cardiovascular outcomes, and mortality in type 2 diabetes. *The New England journal of medicine*.

[51] Marso S. P., Bain S. C., Consoli A. (2016). Semaglutide and cardiovascular outcomes in patients with type 2 diabetes. *The New England journal of medicine*.

[52] Marso S. P., Daniels G. H., Brown-Frandsen K. (2016). Liraglutide and cardiovascular outcomes in type 2 diabetes. *The New England journal of medicine*.

[53] Pouwer M., Heinonen S., Behrendt M. (2016). The APOE^∗^3Leiden.GK+/− mouse as novel translational model for dyslipidemia, type 2 diabetes and macrovascular complications. *Atherosclerosis*.

[54] Kuhnast S., van der Hoorn J. W., Pieterman E. J. (2014). Alirocumab inhibits atherosclerosis, improves the plaque morphology, and enhances the effects of a statin. *Journal of lipid research*.

[55] Kuhnast S., Louwe M. C., Heemskerk M. M. (2013). Niacin reduces atherosclerosis development in APOE^∗^3Leiden.CETP mice mainly by reducing NonHDL-cholesterol. *PLoS One*.

[56] Birjmohun R. S., Hutten B. A., Kastelein J. J., Stroes E. S. (2005). Efficacy and safety of high-density lipoprotein cholesterol-increasing compounds: a meta-analysis of randomized controlled trials. *Journal of the American College of Cardiology*.

[57] Hoogwerf B. J., Bantle J. P., Kuba K., Frantz I. D., Hunninghake D. B. (1984). Treatment of type III hyperlipoproteinemia with four different treatment regimens. *Atherosclerosis*.

[58] Carlson L. A., Oro L. (1973). Effect of treatment with nicotinic acid for one month on serum lipids in patients with different types of hyperlipidemia. *Atherosclerosis*.

[59] Stein E. A., Mellis S., Yancopoulos G. D. (2012). Effect of a monoclonal antibody to PCSK9 on LDL cholesterol. *The New England journal of medicine*.

[60] Evans M., Schumm-Draeger P. M., Vora J., King A. B. (2011). A review of modern insulin analogue pharmacokinetic and pharmacodynamic profiles in type 2 diabetes: improvements and limitations. *Diabetes, obesity & metabolism*.

[61] Hirst J. A., Farmer A. J., Ali R., Roberts N. W., Stevens R. J. (2012). Quantifying the effect of metformin treatment and dose on glycemic control. *Diabetes Care*.

[62] Buse J. B., Henry R. R., Han J., Kim D. D., Fineman M. S., Baron A. D. (2004). Effects of exenatide (exendin-4) on glycemic control over 30 weeks in sulfonylurea-treated patients with type 2 diabetes. *Diabetes Care*.

[63] Nauck M. A., Del Prato S., Duran-Garcia S. (2014). Durability of glycaemic efficacy over 2 years with dapagliflozin versus glipizide as add-on therapies in patients whose type 2 diabetes mellitus is inadequately controlled with metformin. *Diabetes, obesity and metabolism*.

[64] Scott L. J. (2017). Sitagliptin: a review in type 2 diabetes. *Drugs*.

[65] Coghlan M., Leighton B. (2008). Glucokinase activators in diabetes management. *Expert opinion on investigational drugs*.

