# Supplementary material for: The APOE∗3-Leiden Heterozygous Glucokinase Knockout Mouse as Novel Translational Disease Model for Type 2 Diabetes, Dyslipidemia, and Diabetic Atherosclerosis
Source: J Diabetes Res. 2019 Feb 21;2019:9727952. doi: 10.1155/2019/9727952 (PMC6425338; doi:10.1155/2019/9727952)
Supplement: Supplementary Materials — The Supplementary Materials consist of (1) a detailed description of the applied methods and (2) 2 tables: Table I and Table II. Suppl. Table I: biochemical parameters in E3L.GK+/−, E3L, and GK+/− mice. All depicted parameters are measured at week 36, except for liver weight (week 37). Suppl. Table II: plasma cholesterol levels are modulated by the diet in E3L.GK+/− and E3L mice. The response of plasma lipids, glucose, and insulin to the different diets was evaluated. [file 9727952.f1.pdf]

Supplemental material

Methods

Biochemical analyses

Plasma TC and TG were determined throughout the study using enzymatic colorimetric methods (total cholesterol: kit no. A11A01634, Horiba ABX, France and triglycerides: kit no. 12146029, Roche Diagnostics GmbH, Germany) according to the manufacturer's protocols and total cholesterol exposure was calculated. HDL-C was measured after precipitation of ApoB-containing particles(1). The distribution of cholesterol over plasma lipoproteins was determined in groupwise-pooled unfasted sacrifice plasma by fast protein liquid chromatography (FPLC)(2). Blood glucose and HbA_1C levels were measured in one drop of blood obtained from the tail in awake mice, using Accu-Chek (Roche, REF 05599415370) and HbA_1C Now+ (Bayer, REF81611409-3038), respectively, and total glucose exposure was then calculated. Plasma insulin levels were measured with a radioimmunoassay (SRI-13K, Millipore Corporation, USA) on a 1470 Automatic Gamma Counter (PerkinElmer, USA). Plasma ALT and AST were determined using a spectrophotometric assay (Boehringer Reflotron system) in group wise-pooled samples. Urinary albumin and creatinine levels were determined using the mouse albumin ELISA kit (ALPCO, Salem, USA) and the creatinine kit (Exocell, Philadelphia, USA). All assays were performed according to manufacturer's instruction. Hepatic lipid content was analyzed in homogenized, snap-frozen liver samples and analyzed with TINA2.09 software (Raytest Isotopen Meβgeräte, Straubenhardt, Germany).

Histological assessment of atherosclerosis

Hearts fixed in formalin, embedded in paraffin were sectioned perpendicular to the axis of the aorta. Serial cross sections (5 µm thick with intervals of 50 µm) were stained with hematoxylin-phloxine-saffron (HPS) for histological analysis. The average total lesion area per cross section was then calculated(1, 3). For determination of lesion severity the lesions were classified into five categories

according to the American Heart Association classification(4): 0) no lesion, I) early fatty streak, II) regular fatty streak, III) mild plaque, IV) moderate plaque, and V) severe plaque. Lesion composition was determined for the type III-V lesions as a percentage of lesion area after immunostaining with anti-human alpha-actin (1:400; PROGEN Biotechnik GmbH, Germany. Cat#:61001) for smooth muscle cells (SMC), anti-mouse Mac-3 (1:50; BD Pharmingen, the Netherlands. Cat#: 550292) for macrophages and Sirius Red staining for collagen. Necrotic area and cholesterol clefts were measured after HPS staining. Lesion stability index was calculated as described previously(1, 5). In each segment used for lesion quantification, the number of monocytes adhering to the endothelium was counted after immunostaining with AIA 31240 antibody (1:1000; Accurate Chemical and Scientific, New York, New York, USA. Cat#: J1857)(1). Lesion areas were measured using Cell D imaging software (Olympus Soft Imaging Solutions).

Histological assessment of liver steatosis and fibrosis

Liver samples (lobus sinister medialis hepatis) were collected from non-fasted mice, fixed in formalin and paraffin embedded, and sections (3 µm) were stained with haematoxylin and eosin (HE) and Sirius Red. Hepatic steatosis was scored blinded by a board-certified pathologist in HE-stained cross sections using an adapted grading system of human NASH(6, 7). Hepatic fibrosis was identified using Sirius Red stained slides and evaluated using an adapted grading system of human NASH(6, 8), in which the presence of pathological collagen staining was scored as either absent (0), observed within perisinusoidal/perivenular or periportal area (1), within both perisinusoidal and periportal areas (2), bridging fibrosis (3) or cirrhosis (4). In addition, liver fibrosis (expressed as the percentage of the total liver tissue area) was quantified automatically using ImageJ software (version 1.48, NIH, Bethesda, MD, USA)(9).

Histological assessment of diabetic nephropathy

Left kidneys were fixed in formalin, embedded in paraffin and sections (3 µm) were stained with Hematoxyline-Eosin (HE), Masson's trichrome (MTC), Periodic acid–Schiff (PAS) and immunohistochemically for nephrin. Nephrin was stained using a Ventana Discovery with an antibody raised in guinea pig (ab6698, Abcam) diluted 1:1000. Link Rb@GP (Abcam) diluted 1:500, followed by OmniMap@Rb HRP (ROCHE) and ChromoMap DAB-kit (ROCHE) was used to detect the positive reaction. Sections were finally counterstained with HE (ROCHE). An overall score based on the combination of all evaluated parameters was determined blinded by a board-certified pathologist where 0 indicates no change in morphology and 5 indicates severe morphological changes. Vacuolized tubuli were scored as 0 indicating that no vacuolized tubuli are present, 1 indicating small and few vacuoles and 2 indicating large and many vacuoles. Sections stained with HE were evaluated for the presence of renal damage focusing on glomerular damage, including mesangial matrix expansion, and tubule-interstitial damage, including interstitial inflammation, fibrosis and tubular abnormalities, as central causes for loss of kidney function. MTC was used for detection of fibrosis, PAS for scoring of matrix expansion and protein deposition in the tubuli, and nephrin for confirmation of matrix expansion and deletion of nephrin.

References

1. Kuhnast S, van der Tuin SJ, van der Hoorn JW, van Klinken JB, Simic B, Pieterman E, et al. Anacetrapib reduces progression of atherosclerosis, mainly by reducing non-HDL-cholesterol, improves lesion stability and adds to the beneficial effects of atorvastatin. European heart journal. 2015;36(1):39-48.
2. Westerterp M, van der Hoogt CC, de Haan W, Offerman EH, Dallinga-Thie GM, Jukema JW, et al. Cholesteryl ester transfer protein decreases high-density lipoprotein and severely aggravates atherosclerosis in APOE*3-Leiden mice. Arteriosclerosis, thrombosis, and vascular biology. 2006;26(11):2552-9.
3. Delsing DJ, Offerman EH, van Duyvenvoorde W, van Der Boom H, de Wit EC, Gijbels MJ, et al. Acyl-CoA:cholesterol acyltransferase inhibitor avasimibe reduces atherosclerosis in addition to its cholesterol-lowering effect in ApoE*3-Leiden mice. Circulation. 2001;103(13):1778-86.
4. Stary HC, Chandler AB, Dinsmore RE, Fuster V, Glagov S, Insull W, Jr., et al. A definition of advanced types of atherosclerotic lesions and a histological classification of atherosclerosis. A report from the Committee on Vascular Lesions of the Council on Arteriosclerosis, American Heart Association. Arteriosclerosis, thrombosis, and vascular biology. 1995;15(9):1512-31.
5. Kuhnast S, van der Hoorn JW, van den Hoek AM, Havekes LM, Liau G, Jukema JW, et al. Aliskiren inhibits atherosclerosis development and improves plaque stability in APOE*3Leiden.CETP transgenic mice with or without treatment with atorvastatin. Journal of hypertension. 2012;30(1):107-16.
6. Kleiner DE, Brunt EM, Van Natta M, Behling C, Contos MJ, Cummings OW, et al. Design and validation of a histological scoring system for nonalcoholic fatty liver disease. Hepatology (Baltimore, Md). 2005;41(6):1313-21.
7. Liang W, Menke AL, Driessen A, Koek GH, Lindeman JH, Stoop R, et al. Establishment of a general NAFLD scoring system for rodent models and comparison to human liver pathology. PloS one. 2014;9(12):e115922.
8. Tiniakos DG, Vos MB, Brunt EM. Nonalcoholic fatty liver disease: pathology and pathogenesis. Annual review of pathology. 2010;5:145-71.
9. Morrison MC, Mulder P, Salic K, Verheij J, Liang W, van Duyvenvoorde W, et al. Intervention with a caspase-1 inhibitor reduces obesity-associated hyperinsulinemia, non-alcoholic steatohepatitis and hepatic fibrosis in LDLR-/-.Leiden mice. International journal of obesity (2005). 2016;40(9):1416-23.

Tables

Suppl. Table I. Biochemical parameters in E3L. GK$^{+/-}$, E3L and GK$^{+/-}$ mice.

All depicted parameters are measured at week 36, except for liver weight (week 37).

	**E3L.GK$^{+/-}$**	**E3L**	**GK$^{+/-}$**
Weight gain (g)	5 ± 2	5 ± 2	5 ± 2
Weight gain (% of body weight at T=0)	23 ± 6	24 ± 6	24 ± 11
Liver weight (g)	1.9 ± 0.3†	2.0 ± 0.4	1.4 ± 0.3
Liver weight (% of body weight at T=36)	8 ± 1††	8 ± 2	6 ± 1
Cholesterol (mmol/L)	14 ± 3†††	12 ± 3	2 ± 1
Triglycerides (mmol/L)	2.0 ± 0.8†††	1.7 ± 0.4	0.5 ± 0.1
Glucose (mmol/L)	10 ± 1***	8 ± 1	12 ± 2
Insulin (ng/mL)	0.4 ± 0.3	0.1 ± 0.1	0.2 ± 0.1
HbA1c (%)	5.1 ± 0.6**	4.3 ± 0.2	5.4 ± 0.4
Plasma ALT (U/L)	272	199	30
Plasma AST (U/L)	660	402	125
Urinary albumin:creatinin	19 ± 11	16 ± 2	31 ± 34

*** P<0.01, *** P<0.001 when compared to E3L; †P<0.05, †† P<0.01, ††† P<0.001 when compared to GK$^{+/-}$. Data are presented as means ± SD (n = 8-10 per group and insulin n=4-8 per group).*

Suppl. Table II. Plasma cholesterol levels are modulated by the diet in E3L.GK$^{+/-}$ and E3L mice

The response of plasma lipids, glucose and insulin to the different diets was evaluated.

	**Diet at time of plasma sample**	**Chow**	**WTD + 0.15% cholesterol**		**WTD + 0.15% cholesterol and 10% glucose drinking water**			**WTD + 1.0% cholesterol**		
	time (weeks)	*0*	*4*		*8*			*36*		
			mmol/L	relative to T = 0 (%)	mmol/L	relative to T = 0 (%)	relative to T = 4 (%)	mmol/L	relative to T = 0 (%)	relative to T = 8 (%)
Cholesterol	E3L.GK$^{+/-}$	2.6 ± 0.3	6.1 ± 2.1*	143	8.2 ± 2.5**	215	64	14.4 ± 3.0***/†††	463	89
	E3L	3.1 ± 1.5	7.6 ± 1.2**	173	9.0 ± 1.9***	224	20	12.4 ± 2.9***/†	353	43
	GK$^{+/-}$	2.2 ± 0.4	3.2 ± 1.5	60	2.3 ± 0.3	9	-20	2.3 ± 0.8	11	4
Triglycerides	E3L.GK$^{+/-}$	2.2 ± 0.3	1.5 ± 0.7	-30	2.3 ± 0.9	3	95	2.0 ± 0.8	-14	-5
	E3L	2.1 ± 0.3	2.2 ± 0.6	5	3.0 ± 1.7	45	45	1.7 ± 0.4	-15	-28
	GK$^{+/-}$	0.7 ± 0.0	0.7 ± 0.4	-2	0.6 ± 0.1	-14	1	0.5 ± 0.1	-33	-19
Glucose	E3L.GK$^{+/-}$	12.7 ± 1.8	12.8 ± 3.6	1	13.0 ± 1.7	4	7	10.4 ± 1.4	-19	-17
	E3L	7.7 ± 0.7	9.7 ± 1.5*	26	8.5 ± 1.1	11	-10	8.0 ± 1.2	5	-4
	GK$^{+/-}$	13.3 ± 2.6	14.7 ± 2.5	14	12.4 ± 2.4	-6	-14	14.1 ± 2.6	9	17
Insulin	E3L.GK$^{+/-}$	0.3 ± 0.1	0.4 ± 0.1	30	0.4 ± 0.2	43	8	0.4 ± 0.3	-13	9
	E3L	0.4 ± 0.1	0.4 ± 0.2	15	0.4 ± 0.0	7	3	0.1 ± 0.1*/†	-60	-66
	GK$^{+/-}$	0.4 ± 0.1	0.4 ± 0.1	3	0.5 ± 0.4	32	24	0.2 ± 0.1	-42	-41

** P<0.05, ** P<0.01, *** P<0.001 when compared to T = 0 weeks; †P<0.05, ††† P<0.001 when compared to previous time point.*
Data are presented as means ± SD (n = 8-10 per group and insulin n=4-8 per group). WTD, western type diet.
